# Extremely Small Clutch Size and Juvenile Survival in a Social Cichlid of an African Great Lake

**DOI:** 10.1002/ece3.72497

**Published:** 2025-11-10

**Authors:** Shun Satoh, Yuki Yoshio

**Affiliations:** ^1^ The Hakubi Center for Advanced Research Kyoto University Kyoto Japan; ^2^ Department of Zoology, Graduate School of Science Kyoto University Kyoto Japan

## Abstract

*Neolamprologus buescheri* , a cooperatively breeding cichlid fish endemic to Lake Tanganyika, exhibits one of the slowest life histories documented among teleost fish. With remarkably small clutch sizes ranging from one to seven eggs (median = 3) and exceptionally high juvenile survival rates of over 50% within two months, this species deviates significantly from typical fish reproductive patterns. Its slow somatic growth, prolonged parental care, and occupation of deep‐water habitats place it at the extreme end of the fast–slow life‐history continuum among fish.

## Graphical abstract

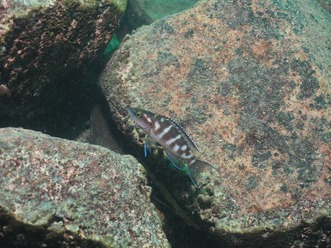

Understanding the remarkable diversity of life histories among organisms is a key objective in evolutionary biology. The life histories of animals, shaped by underlying demographic processes, tend to exhibit characteristic patterns within taxonomic groups. The fast–slow continuum, defined by traits such as age at maturity, lifespan, survival rate, reproductive rate and population growth rate, offers a theoretical framework for understanding the diversity of life histories and their evolutionary trajectories (Roff 2002; Jeschke et al. 2008).

Among vertebrates, teleost fish are generally characterized by a fast life history strategy. They reach sexual maturity rapidly, have short lifespans and demonstrate high fecundity, often accompanied by exceptionally high mortality rates in the early stages of life (Kamler 2012). However, in contrast to this general tendency in fish life histories, some species have evolved reproductive strategies to avoid high mortality rates during the early stages of life. All species in the Cichlidae family, for example, exhibit some form of parental care, using strategies such as brood care, food provisioning and cooperative breeding to mitigate the ecological challenges associated with this critical period (Keenleyside 1991). In the present study, we highlight *Neolamprologus buescheri* , an endemic species of Lake Tanganyika in Africa, as a prime example of a species with a slow life history (Figure 1). *Neolamprologus buescheri* is remarkable not only among all teleost fishes but also within the cichlids, which include numerous species with slow life histories.

**FIGURE 1:**
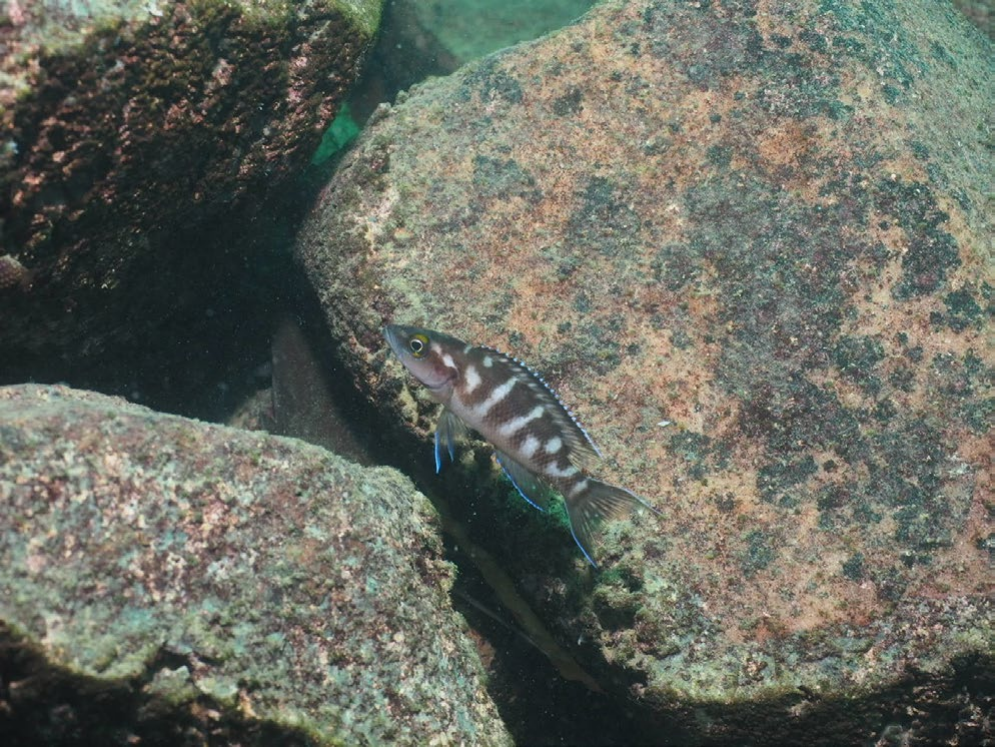
Breeding female of *Neolamprologus buescheri* in their breeding territory. Photograph taken by S.S.

*Neolamprologus buescheri* is a tiny cooperative breeding cichlid, and their body size is ca. 75 mm SL (Standard length) in breeding males and ca. 55 mm in breeding females (Figure 2a) (Satoh et al. 2025). This species occupies the profound recesses of the reef zones of the lake's deep water and constructs breeding nests within diminutive rock crevices. This species is widely distributed across the lake and exhibits notable regional color variation (Koning 1998); however, knowledge of its natural history and ecology remains limited.

**FIGURE 2:**
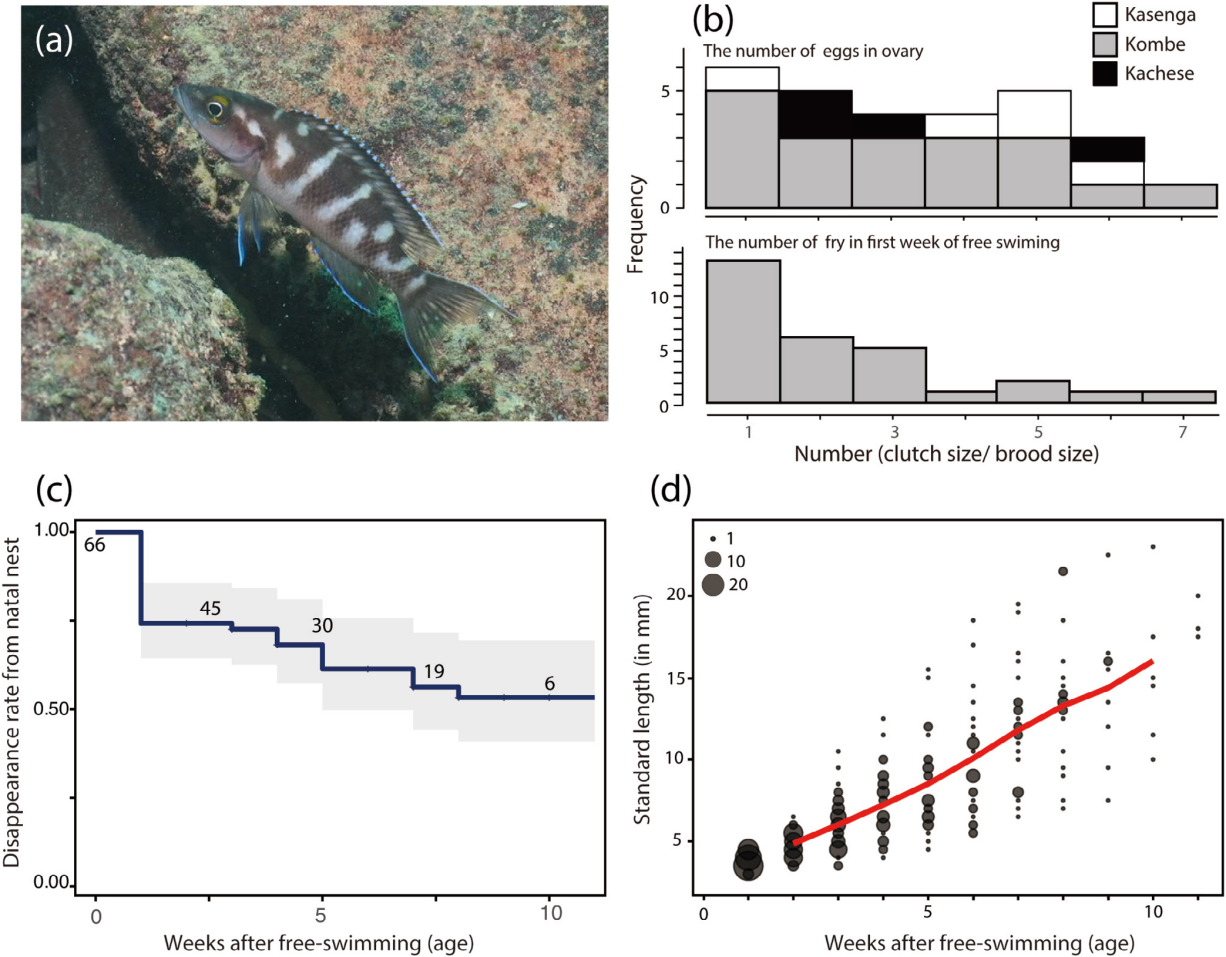
Exceptionally small clutch size, high survival, and slow growth during early life in *Neolamprologus buescheri* , a species endemic to Lake Tanganyika. (a) A breeding female inhabits a deep‐water rocky reef. (b) Clutch size (number of eggs in the ovary) and brood size (number of fry in the first week of free‐swimming per breeding nest). Clutch size data were collected from three populations (white: Kasenga; gray: Kombe; black: Kachese), while brood size was recorded at Kombe Point. (c) Relationship between the per‐fry disappearance rate from the breeding nest and age (weeks after free‐swimming). Solid lines represent Kaplan–Meier survival curves with 95% confidence intervals; numbers indicate risk tables. (d) Relationship between body size (standard length) and age (in weeks post free‐swimming; fry begin free‐swimming roughly five days after hatching). The red line indicates the moving average. The plot size represents the number of fry.

According to limited field research (Satoh et al. 2025), female breeders defend their offspring from predators. As juveniles grow, they do not disperse from the breeding nest but actively engage in brood and territorial defense as helpers. Therefore, the breeding group of *N. buescheri* typically comprises up to four helpers, a breeding female, and fry (Satoh et al. 2025). Furthermore, the mating system of this species is polygynous, with multiple groups of breeding females occurring within the home range of a single breeding male (Satoh et al. 2025).

Our field sampling of *N. buescheri* revealed that the number of mature eggs in the ovaries of female breeders ranged from 1 to 7 (median = 3, *n* = 28 females from three populations). Remarkably, 21.4% of female breeders (*n* = 6) had only one egg in their ovary (Figure 2b). The number of fry per breeding nest (brood size) immediately after the onset of free‐swimming was also 1–7 fry (median = 2 fry, *n* = 29 breeding nests), which was almost consistent with the number of eggs in the ovaries (Figure 2b). These findings strongly suggest that this species has a markedly diminished number of eggs per spawning event.

Additionally, despite the tiny clutch/brood size, fry of *N. buescheri* survival is exceptionally high (Figure 2c). Follow‐up observations conducted for up to 11 weeks in Lake Tanganyika revealed that over 50% of offspring remained within the breeding nest after two months (*n* = 64 juveniles from 29 nests). This figure may, in fact, underestimate true juvenile survival, as subordinate juveniles are frequently expelled from their natal nests due to aggressive interactions with dominant siblings (Satoh et al. 2021). In other words, during the early life history stages—when most fishes experience their highest levels of predation—this species maintains a survival rate of at least 50% within the first two months, a rate comparable to, or even exceeding, that reported for chimpanzee infants (Nishida et al. 2003), for example.

The exceptional survival of this species is likely due to a combination of social and ecological factors. Notably, they are a cooperative breeding species, meaning offspring are protected from predators by not only parents, but also group members (Tanaka et al. 2018). Their distinctive social structure, which promotes communal living, is likely to contribute significantly to their remarkable juvenile survival rates. Additionally, this species may have substantially enhanced its survival rate by markedly reducing feeding activity or metabolic expenditure (Werner and Anholt 1993).

Therefore, we examined the growth pattern of the fry in this species (Figure 2d). During the first 10 weeks after free‐swimming, fry reached an average size of approximately 15.25 ± 4.2 mm SL (mean ± SD), representing an exceptionally slow growth rate relative to other cichlid species reported in previous field studies (Nagoshi and Yanagisawa 1997); Nagoshi and Yanagisawa (1997) reported that growth rates during the early period ranged from 0.25 to 0.47 mm SL per day from 12 cichlid species in Lake Tanganyika, while this species is about ca. 0.18 mm SL per day. In the fast–slow continuum concept for life history, growth and survival are often viewed as a trade‐off. Fry of this species are known to spend most of their time in rock shelters within the breeding territory of the parents (Satoh et al. 2025). In addition to the generous protection provided by the cooperative breeding group, reduced feeding activity may be responsible for the remarkably high survival rate in this species.

## 1 Methods

### 1.1 Field Sampling and Observation

Field sampling and behavioral observations were conducted via scuba diving at three sites located at the southern end of Lake Tanganyika, the Republic of Zambia: Kasenga (8.71530° S, 31.14182° E), Kombe (8.79839° S, 31.02001° E), and Cape Kachese (8.49187° S, 30.47987° E). Of these locations, Kombe Point was prioritized for sampling and observation as *N. buescheri* occurs there in relatively shallow waters, facilitating easier capture. All experimental procedures were approved by the Animal Care and Use Committees of the Graduate School of Advanced Studies at Kyoto University and were conducted in accordance with the ASAB/ABS Guidelines for the Use of Animals in Behavioral Research. Fieldwork in Lake Tanganyika was carried out with authorization from the Zambian Ministry of Agriculture, Livestock and Fisheries, and was conducted in full compliance with Zambian regulations.

### 1.2 Measurements of Life History Traits

To determine the clutch size of *N. buescheri* in its natural habitat, we haphazardly captured breeding females from three populations using fine‐mesh gill nets and hand nets: five individuals from Kasenga (depth: 25–35 m), 19 from Kombe (depth: 9–25 m), and four from Kachese Point (depth: 23–28 m). All sampling sites were rocky habitats, situated approximately 16–80 km apart from one another. However, owing to limited knowledge of genetic structure in this species, it remains unclear whether these populations are genetically isolated. Breeding females exhibit territorial defense behavior toward conspecifics and other species (Satoh et al. 2025). After confirming through direct behavioral observations that the focal individuals occupied specific shelters and displayed aggressive responses toward intruders, the observer captured them in Kasenga and Kachese points. With regard to Kombe point, we utilized data exclusively from individuals previously identified as breeding females through behavioral observations conducted by the authors (Satoh et al. 2025). Following capture, the fish were euthanized using an overdose of tricaine methanesulfonate (MS‐222, Sigma Chemical Co.) and subsequently preserved in 10% formalin. Standard length (SL, mm), body weight (BW, mg), gonad weight (GW, mg), and clutch size (the number of mature eggs in the ovary) were measured; however, only the clutch size data are reported in this study.

### 1.3 Survival and Growth Rate of Fry

The neonatal period represents a critical time window that can profoundly influence an individual's fitness. Accordingly, we assessed survival and growth rates of this species during the initial post‐hatching phase. A total of 29 breeding nests were identified, collectively containing 66 fry at the onset of free‐swimming. Fry were classified as newly free‐swimming when their SL ranged between 3.5 and 4.5 mm. Breeding groups were identified by placing numbered stones in their vicinity, and the fish remained in the same locations throughout the study period. As individual identification of fry was not feasible, survival rates were estimated by conducting weekly nest‐based fry counts. To evaluate growth, the SL of all fry was measured to the nearest 0.5 mm using calipers during weekly monitoring. This was facilitated by the species' tendency to remain stationary on rock surfaces when approached by observers: the fry's body size was measured by carefully bringing the caliper as close as possible to the immobile fry on the rock surface. In these observations, juveniles, helpers, and parents were not captured to avoid disturbing fry survival and growth. Breeding nests and associated fry were monitored for up to 11 weeks, although some observations were terminated earlier due to constraints in the research trip schedule.

### 1.4 Visualization of Life History Traits

Since the objective of this study was to document the specific life history traits of *N. buescheri* , no statistical comparisons were conducted. The R scripts (R Core Team 2023) used to generate the figures presented in the main text, along with the complete dataset—including data, such as SL, BW and GW, not featured in the manuscript—are available in Dryad (Satoh and Yoshio 2025). To illustrate survival rates, the Kaplan–Meier survival curve was employed. Accordingly, the weekly brood size data were transformed into a format representing the number of weeks each individual was observed within the nest. Moreover, whether the termination of observations was due to the disappearance of fry or the conclusion of the study period was also recorded to accurately depict the survival curve.

## 2 Conclusion

*Neolamprologus buescheri* , which inhabits the deep waters of the African Great Lakes, represents an exceptional case on the fast–slow continuum of life history among fish. It produces a minimum of one egg, with early‐life survival exceeding 50%, yet its growth rate is exceptionally slow compared to other cichlids in Lake Tanganyika. Its life history may be the slowest ever recorded in a fish, reshaping our understanding of the evolution of vertebrate life histories. This case study highlights the importance of using African Great Lakes cichlids as model systems for investigating the evolution of extreme reproductive strategies.

## Author Contributions

Shun Satoh: conceptualization (lead), funding acquisition (lead), methodology (lead), project administration (lead), visualization (lead), supervision (lead), writing – original draft (lead), writing – review and editing (lead). Yoki Yoshio: formal analysis (lead), writing – original draft (supporting), writing – review and editing (supporting).

## Conflicts of Interest

The authors declare no conflicts of interest.

## Data Availability

As outlined in the main text, all the data that support our findings, including the R code used for visualization, can be found in Dryad (DOI: https://doi.org/10.5061/dryad.83bk3jb59).
